# CLINICAL EFFECTIVENESS OF ENDOSCOPIC SUBMUCOSAL DISSECTION IN THE MANAGEMENT OF SUPERFICIAL ESOPHAGEAL NEOPLASMS ASSOCIATED WITH BARRETT’S ESOPHAGUS

**DOI:** 10.1590/S0004-2803.24612025-033

**Published:** 2026-03-02

**Authors:** Rúbia Moresi VIANNA DE OLIVEIRA, Josué ALIAGA RAMOS, Jonathan Richard WHITE, Vitor Nunes ARANTES

**Affiliations:** 1 Hospital Mater Dei Contorno, Endoscopy Unit, Belo Horizonte, MG, Brazil.; 2 Hospital José Agurto Tello-Chosica, Department of Gastroenterology; Clinica Madre Zoraida, Service of Gastroenterology, Lima, Perú.; 3 University of Nottingham, Nottingham University Hospitals NHS Trust, Nottingham Digestive Diseases Centre and NIHR Nottingham Biomedical Research Centre, Nottingham, UK.; 4 Hospital Mater Dei Contorno, Federal University of Minas Gerais, Endoscopy Unit, Alfa Institute of Gastroenterology, Belo Horizonte, BH, Brazil.

**Keywords:** Barrett esophagus, esophageal neoplasms, endoscopic mucosal resection, Esôfago de Barrett, neoplasias esofágicas, ressecção endoscópica da mucosa

## Abstract

**Background:**

The main clinical impact of superficial neoplasms associated with Barrett’s esophagus lies in their increasing oncogenic potential in the medium and long term. For this reason, the main international guidelines agree on the importance of their early eradication. However, controversy persists as to the most appropriate endoscopic resection technique either endoscopic mucosal resection or endoscopic submucosal dissection (ESD) that guarantees the best resective quality standards.

**Objective:**

This study aims to present the results of the clinical application of endoscopic submucosal dissection to manage superficial esophageal neoplasms in Barrett’s esophagus.

**Methods:**

A retrospective analysis was performed on a prospectively collected database on consecutive patients treated with ESD for superficial neoplasms associated with Barrett’s esophagus, between 2009 and 2022. The following clinical outcomes were assessed: en-bloc, complete and curative resection rates, local recurrence, adverse events and procedure-related mortality.

**Results:**

Esophageal ESD was carried out in 27 patients with a final histological diagnosis of adenocarcinoma in 55.6% and high-grade intraepithelial neoplasia in 44.4%. En bloc and complete resection rates were 96.2% and 85.1%, respectively. The curative resection rate was 77.7%. Adverse events occurred in two cases (7.4%). The mean post ESD endoscopic follow up was 22.1 months. Disease free survival rate at 2 years was 88.9%.

**Conclusion:**

ESD performed by trained endoscopists is feasible, safe and clinically effective for managing early Barrett’s esophagus neoplasm.

## HIGHLIGHTS

• Endoscopic submucosal dissection (ESD) in the management of superficial neoplasms associated with Barrett’s esophagus has the potential to replace endoscopic mucosal resection (EMR) as the first-line treatment for the eradication of this type of lesion.

• ESD in the management of superficial neoplasms associated with Barrett’s esophagus show high rates of en bloc resection regardless of lesion size.

• ESD in the management of superficial neoplasms associated with Barrett’s esophagus show low rates of adverse events in expert hands.

• Our study shows one of the largest audits of ESD in the management of superficial neoplasms associated with Barrett’s esophagus in Latin American centres.

## RESUMO

**Contexto:**

O principal impacto clínico das neoplasias superficiais associadas ao esôfago de Barrett reside no seu crescente potencial oncogênico a médio e longo prazo. Por esse motivo, as principais diretrizes internacionais concordam com a importância de sua erradicação precoce. No entanto, persiste a controvérsia quanto à técnica de ressecção endoscópica mais adequada, seja a ressecção endoscópica da mucosa ou a dissecção endoscópica da submucosa (ESD), que garanta os melhores padrões de qualidade ressecativa.

**Objetivo:**

Este estudo tem como objetivo apresentar os resultados da aplicação clínica da dissecção endoscópica da submucosa no tratamento de neoplasias esofágicas superficiais no esôfago de Barrett.

**Métodos:**

Foi realizada uma análise retrospectiva de um banco de dados prospectivamente coletado de pacientes consecutivos tratados com ESD para neoplasias superficiais associadas ao esôfago de Barrett, entre 2009 e 2022. Foram avaliados os seguintes desfechos clínicos: taxas de ressecção en-bloc, completa e curativa, recorrência local, eventos adversos e mortalidade relacionada ao procedimento.

**Resultados:**

A ESD esofágica foi realizada em 27 pacientes, com diagnóstico histológico final de adenocarcinoma em 55,6% e neoplasia intraepitelial de alto grau em 44,4%. As taxas de ressecção em bloco e completa foram de 96,2% e 85,1%, respectivamente. A taxa de ressecção curativa foi de 77,7%. Ocorreram eventos adversos em dois casos (7,4%). O acompanhamento endoscópico médio pós-ESD foi de 22,1 meses. A taxa de sobrevida livre de doença em 2 anos foi de 88,9%. **Conclusão:** A ESD realizada por endoscopistas treinados é viável, segura e clinicamente eficaz para o tratamento da neoplasia precoce do esôfago de Barrett.

## INTRODUCTION

Barrett’s esophagus (BE) is defined as intestinal metaplasia of the distal esophagus, in which the squamous epithelium is replaced by columnar epithelium with goblet cells. This is a condition that is associated with gastroesophageal reflux disease (GERD), and approximately 5% to 12% of patients with chronic GERD symptoms develop BE. BE is a known precursor of esophageal adenocarcinoma, a highly lethal cancer with an increasing incidence over the past five decades and a 5-year survival of less than 20%^1^ ^-^ ^4^.

Endoscopy plays a crucial role in the detection and treatment of early Barrett’s esophagus neoplasm (EBEN). When detected early and treated endoscopically, this confers a much more favourable prognosis with a more precise histological diagnosis and potentially curative resection. One of the great benefits of endoscopic submucosal dissection (ESD) in the eradication of superficial neoplasms in Barrett’s Esophagus is its high rate of en bloc resection regardless of the size of the lesion. This allows for more precise histopathological analysis of the resected specimen which is better for oncological staging, thus reducing the interobserver variability^5^ ^-^ ^9^.

ESD in Barrett’s esophagus shows optimal outcomes similar to those obtained with traditional surgery but mains the affected structure and avoids post-surgical morbidity associated with esophagectomy. However, one of the major limitations of this procedure is the high level of technical complexity required for its optimal execution^10^ ^-^ ^15^. The aim of our study is to present the outcomes of a large series of patients with neoplasms associated with Barrett’s esophagus managed by ESD.

## METHODS

### Patients

This is a retrospective observational study. The data was extracted from a prospectively generated database including consecutive patients from 2009 to 2022, that underwent ESD for early Barrett’s esophagus neoplasm. Inclusion criteria were all participants referred for endoscopic resection (ER) with EBEN larger than 15 mm in size were included. All lesions were assessed by image-enhanced endoscopy, which included high-resolution white light endoscopy, digital chromoendoscopy including narrow band imaging (NBI), Fuji intelligent chromoendoscopy, linked colour imaging, and blue laser imaging. In addition, magnifying endoscopy and chromoendoscopy with acetic acid was utilized in the preoperative assessment to optimize lesions characterization. Depending on clinical need, in selected cases computed tomography and endoscopic ultrasonography were performed for preoperative staging. We excluded patients with clinical conditions unsuitable for general anaesthesia, or with advanced tumours.

The following data were collected: Age, sex, preoperative biopsy findings, lesion size, Paris endoscopic classification, procedure duration, resection histology, local recurrence, metachronous lesions, adverse events, and hospital admissions. En bloc resection rate, complete resection rate with free margins (R0) and curative resection rate were calculated according to current European guidelines^1^. Resection of the tumour in one piece rate was defined as en bloc resection, complete tumour resection rate with free margins of neoplasia was defined as R0 resection. The curability criteria for adenocarcinomas were considered as follows.

Very low risk of lymph node metastasis: Complete and en bloc resection; tumors limited to the epithelium (pT1a-EP), lamina propria (pT1a-LMP), or muscularis mucosa (pT1a-MM); absence of lymphovascular invasion, and well-to-moderate grade of differentiation.

Low risk of lymph node metastasis: Complete and en bloc resection; tumours with superficial submucosal invasion (pT1b-Sm1, ≤500 μm); absence of lymphovascular invasion; and well-to-moderate grade of differentiation.

Patients with lesions demonstrating deep submucosal invasion (SM2) on histological assessment (T1b stage), or with the presence of lymph-vascular invasion or compromised margins were defined as non-curative resection.

Metachronous and local recurrence rate lesions were analyzed during post procedure endoscopic follow-up. Endoscopic follow-up was scheduled at three months, and then annually. Local recurrence was characterized as the emergence of an esophageal neoplastic lesion at the identical site of a prior resection during endoscopic surveillance. In contrast, a metachronous lesion was defined as the development of a new esophageal lesion in a different anatomical location from the primary resection, occurring after a minimum follow-up period of six months. Furthermore, if a subsequent esophageal tumour was identified within six months of the initial resection, it was classified as a missed lesion.

### ESD procedures

**FIGURE 1:**
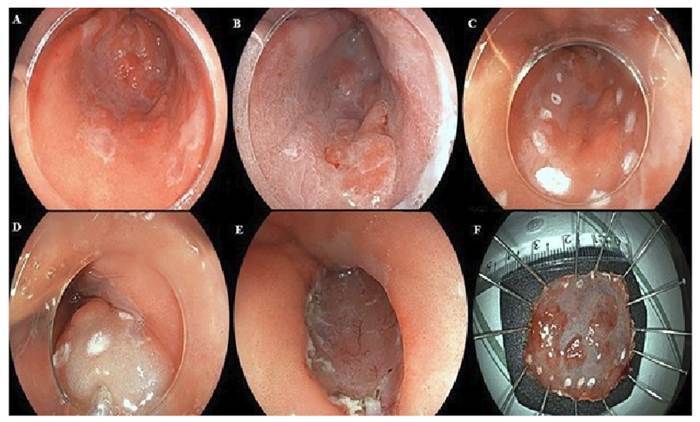
Illustrative images of ESD in superficial esophageal neoplasms in Barrett’s esophagus. 1A: superficial esophageal neoplasm in Barrett’s esophagus visualized with high-resolution white light endoscopy. 1B: superficial esophageal neoplasm in Barrett’s esophagus visualized with chemical chromoendoscopy with acetic acid. 1C: marking placement. 1D: submucosal injection. 1E: final defect post ESD. 1F: resected specimen with en bloc resection.

The procedure was performed by a single trained endoscopist (VA) under general anaesthesia. Each procedure was comprised of the following sequential steps: (1) Marking placement in soft coagulation mode; (2) Submucosal injection with 25-gauge needles to lift the lesion utilizing a 0.4% sodium hyaluronate solution in a teardrop configuration (Adaptis Fresh; Legrand Laboratory, Campinas, Brazil)^16^; (3) Mucosal incision using Endocut I mode; (4) Submucosal dissection and trimming with forced coagulation; and (5) Vessels coagulation, either with a knife or coagulation forceps. Intravenous antibiotic prophylaxis with cephalosporin (or clindamycin in penicillin allergy) was provided to all patients^17^. Figure 1 shows illustrative endoscopic images of a representative case of ESD in superficial esophageal neoplasms in Barrett’s esophagus.

During the postoperative period, proton pump inhibitors and sucralfate was administered for four and two weeks respectively. Additionally, a prednisone-based regimen was administered over four weeks to patients who underwent semi-circumferential resection involving more than 75% of the circumference^18^.

### Statistical analysis

Data were organized and recorded using Microsoft Excel Windows 2010 (Microsoft Corp., Redmond, WA, United States). A descriptive statistical analysis was conducted, which included the calculation of frequency and proportion for categorical variables, assessment of average values, SD, median, and mean±SD for continuous variables.

### Ethical considerations

This retrospective audit received Institutional Review Board (IRB) approval (number 23072.201037/ 2022-29) in 2022.

## RESULTS

ESD was undertaken on a total of 27 patients (26 men, 96.30%) with a mean age of 66 years. The mean lesion size was 35.96 mm (±16.82), with the majority classified as Paris 0-IIa (48.14%) and 0-IIb (33.34%). In the pre-procedural histological evaluation, 51.85% (14/27) were classified as high-grade dysplasia and 48.15% (13/27) as adenocarcinoma. Table 1 demonstrates the clinical and pathological features of the patients. The average duration of the procedures was 132.4 minutes (±60.24), and the average size of the resection site measured 6.04 centimetres (±1.96). Notably, 18.51% (5/27) of the resections involved more than 75% of the esophageal circumference. En bloc resection was successfully accomplished in 96.29% (26/27) of the cases.

**TABLE 1.** Patients and lesions characteristics.

Number of patients/lesions	27/27
Age years, mean+SD	66.77+9.02
Gender (male/female)	26 (96.30)/1 (3.70)
Paris classification	
0-Ip	0 (0)
0-Is	3 (11.10)
0-IIa	13 (48.14)
0-IIb	9 (33.30)
0-IIc	2 (7.40)
0-III	0 (0)
Pre-ESD biopsy	
High grade dysplasia	14 (51.80)
Adenocarcinoma	13 (48.10)
Procedure time (minutes), mean+SD	132.40+60.24
Circumferential extent of the resection >75% of the circumference (%)	5 (18.50)
Maximum diameter of the lesion mm, (median)	35.90 (30.00)
Size of the resection (cm^2^)	6.04

Esd: endoscopic submucosal dissection.

Histological analysis conducted post-resection revealed that 44.40% of the lesions were classified as high-grade intraepithelial neoplasia, while 55.60% were identified as adenocarcinoma. A total of 40.70% (11/27) of the specimens revealed neoplasia confined to the mucosal layer (M1). 77.77% of the cases (21/27) were well differentiated. A significant histological discrepancy between the preoperative biopsy and the pathological examination of the resected specimens was observed in 23.30% of the patients. The R0 resection rate was found to be 85.18% (23/27), and the curative resection rate was 77.77% (21/27). The clinical outcomes of ESD are summarized in Table 2.

**TABLE 2:** Clinical outcomes of endoscopic submucosal dissection in early Barrett^,^ s esophagus neoplasms.

En bloc resection (n=27)	26 (96.2)
Complete resection (R0) (n=27)	23 (85.1)
Curative resection (n=27)	21 (77.7)
Adverse events	
Acute adverse events	1 (3.7)
Late adverse events	1 (3.7)
Bleeding	0 (0)
Perforation	0 (0)
Esophageal stricture	1
Local recurrence	3 (11.1)
Metachronous lesions	5 (18.5)
Infiltration to deep layers	
T1a M1	11 (40.7)
T1a M2	3 (11.1)
T1a M3	4 (14.8)
T1b Sm1	4 (14.8)
T1b Sm2	5 (18.5)
Degree of cell differentiation	
Well differentiated	21 (77.7)
Moderately differentiated	3 (11.1)
Poorly differentiated	3 (11.1)
Histological analysis of the resected specimen	
High grade dysplasia	12 (44.4)
Adenocarcinoma	15 (55.5)
Lymphovascular invasion	2 (7.4)
Correspondence between pre and post ESD biopsies	21 (77.7)
Length of hospital stay days, mean ± SD	2.40 ± 0.91
Follow-up time months, median	22.1 (12.0)

Adverse events occurred in two patients. One patient developed an intraoperative cardiac arrythmia which was treated and did not preclude the ESD procedure. This patient remained in the intensive care unit (ICU) for postoperative observation for 2 days and later was discharged after cardiology review. Another patient developed bacteraemia and sepsis on postoperative day 1 and was transferred to ICU. They were managed with antibiotics and was discharged on postoperative day 4. The mean post procedure hospitalization period was 2.40 days (±0.91).

During a mean follow-up of 22.18 months, one patient developed an esophageal stricture and was treated successfully with endoscopic dilation. The rates of local recurrence and metachronous lesions were 11.1% and 18.5%, respectively. These were managed with endoscopic resection or ablation therapy. One patient required radiofrequency ablation and two patients underwent argon plasma coagulation ablation for remaining BE epithelium. No patients were referred for surgery and there was no procedure-related mortality.

## DISCUSSION

ESD has been increasingly utilized for endoscopic resection of superficial neoplastic lesions in the gastrointestinal tract, enabling higher rates of en bloc and curative resections compared to endoscopic mucosal resection (EMR), particularly for lesions over 2 cm in size. In this study, ESD was a safe and effective therapeutic modality for managing patients with EBEN, achieving high rates of en bloc and curative resections, with low risk of complications^19^ ^-^ ^23^. However, for patients with EBEN, EMR remains the most utilized ER method currently^19^ ^,^ ^24^ ^-^ ^27^.

The main limitation of EMR is the fragmented nature of resection with larger lesions greater than 15 mm in size. Piece-meal EMR can limit histopathological analysis and impair an accurate assessment of tumour resection curability. Fragmented EMR is therefore, considered a risk factor for recurrence^28^ ^-^ ^30^. In contrast, ESD offers higher en bloc resection rates regardless of lesion size. In this study, with a mean lesion size of 35.9 mm an en bloc resection rate of 96.2% was achieved, providing supporting evidence that ESD is an effective method for en bloc resection of large lesions^7^ ^,^ ^21^ ^,^ ^24^.

The main challenges in the endoscopic approach to Barrett’s esophagus-associated neoplasms include the difficulty in achieving precise delineation of the lateral margins of the lesion within Barrett’s mucosa. However, innovative image-enhancing endoscopic techniques applied by experts have overcome this limitation. Another factor that increases the technical complexity during ESD in superficial neoplasms associated with this clinical condition is the increased thickness of Barrett’s mucosa due to a double mucosal effect present in this intestinal metaplasia. In this scenario, the experience of the operator and the adequate choice of the resective technique is fundamental to overcome this^26^ ^-^ ^28^. In our case series, we achieved high rates of en bloc and complete resection, even in highly challenging neoplasms. Many of our non-curative cases were mainly due to oncologic progression of the neoplasm.

There was significant histological discrepancy between preoperative biopsy and resection histology in 23.3% of patients. In the preoperative biopsy, 14 (51.8%) patients had high grade dysplasia and 13 (48.1%) had adenocarcinoma. In the analysis of the resected specimen, this proportion was reversed with 12 (44.4%) of the patients having high-grade dysplasia and 15 (55.5%) adenocarcinoma (Table 3). This level of disagreement amongst pre and post histological findings has previously been observed^3^ ^,^ ^20^ ^,^ ^21^ ^,^ ^31^ ^-^ ^33^. Larger histological specimens provided by ESD have been shown to reduce interobserver variability associated with EBEN evaluation by pathologists. Studies comparing the frequency of histological diagnosis changes between EMR and pre-EMR biopsy diagnosis for patients with EBEN have demonstrated that endoscopic resection resulted in a diagnostic change for approximately 31% to 49% of patients. Podboy et al. demonstrated that EMR was associated with greater pathological uncertainty in Barrett’s neoplasia, when compared to ESD due to ESD having more R0 resections and en bloc resections when compared to EMR. EMR also shows significantly higher rates of lateral margin involvement (13/31, 41.9% vs 1/20, 5.0%) and vertical margin involvement (13/31, 41.9% vs 0/20, 0.0%), when compared to ESD. This led to an inability to reach a definitive diagnosis in 13/31 EMR specimens compared to 0/20 ESD specimens (*P*=0.003). Furthermore, compared to EMR, patients with BE with dysplasia or intramucosal adenocarcinoma undergoing ESD achieved higher rates of complete dysplasia remission with similar complication rates between the two groups^8^. In summary, both EMR and ESD can provide information for the pathological staging of early esophageal adenocarcinoma, and specifically for deep margin involvement, but ESD provides more precise information^8^ ^,^ ^22^ ^,^ ^23^.

**TABLE 3:** Histological analysis in preoperative biopsy and specimen histology.

Pre-ESD biopsy	
High grade dysplasia	14 (51.8)
Adenocarcinoma	13 (48.1)
Histological analysis of the resected specimen	
High grade dysplasia	12 (44.4)
Adenocarcinoma	15 (55.5)

ESD: endoscopic submucosal dissection.

One limitation of ESD compared to EMR is the procedure time. In the present analysis, the mean procedure time was 132.4 minutes, which is comparable to other studies that evaluated esophageal ESD^34^ ^-^ ^37^. The clinical effectiveness of ESD in treating EBEN in this study was remarkable, with an R0 resection rate of 85.18% and a curative resection rate of 77.77%, along with a low rate of local recurrence and the need for surgical treatment, 11.1% and 0.0%, respectively. Similar findings were reported by Yang et al. in a meta-analysis that demonstrated an ESD R0 and curative resection rate of 74.5% and 64.9% for EBEN, respectively^12^. Another limitation for ESD application for EBEN in clinical practice is the high level of skill required. However, experience with ESD is increasing in the Western world and with greater user skill procedure length and complication rates should reduce. 

In this series, the two adverse events were not directly related to the ESD procedure. No intraoperative hemorrhage, perforation or mortality occurred in this series. The incidence of these adverse events is comparable to the reports from previous studies. We experienced only one late complication (esophageal strictures) that were managed successfully by endoscopic balloon dilation^36^ ^-^ ^39^.

Our study limitations included the fact that the number of patients included was relatively small compared to prior studies conducted in Asia. However, to our knowledge, this represents one of the largest case series reported from Latin America, with clinical outcomes comparable to those observed in leading European centers. Another limitation was that all procedures were performed by a single endoscopist at two referral endoscopy centers in Brazil. They were both highly skilled after receiving specialized training in high-volume Japanese institutions. As a result, a high degree of technical consistency and procedural standardization was demonstrated but it may limit the generalizability of our findings to other endoscopy units with differing levels of expertise, infrastructure and training backgrounds.

## CONCLUSION

Our study provides further data that ESD should be considered as an important therapeutic option for managing superficial neoplasms associated with Barrett’s esophagus. It is safe and effective, achieves high rates of curative, en bloc and complete resection with low adverse event rates in experts hand. This procedure has the potential to become first line treatment for this type of neoplastic lesions.

## Data Availability

Data available in the article: Results section (Tables 1, 2, and 3)
